# APOB is a potential prognostic biomarker in hepatocellular carcinoma

**DOI:** 10.1007/s12672-024-00877-6

**Published:** 2024-02-03

**Authors:** Zhifeng Lin, Xiaohui Ji, Nana Tian, Yu Gan, Li Ke

**Affiliations:** 1 Department of Medical Record; Guangdong Provincial Clinical Research Center for Obstetrics and Gynecology; Guangdong Provincial Key Laboratory of Major Obstetric Diseases, The Third Affiliated Hospital of Guangzhou Medical University, Guangzhou, China; 2 Department of Obstetrics and Gynaecology, Sun Yat-Sen Memorial Hospital, Sun Yat-Sen University, Guangzhou, China; 3 Department of Medical Record, The Fifth Affiliated Hospital of Guangzhou Medical University, Guangzhou, China

**Keywords:** Hepatocellular carcinoma, NcRNAs, Prognosis, Expression, APOB

## Abstract

Hepatocellular carcinoma (HCC) is significantly associated with adverse prognostic outcomes. The development and progression of different types of human tumors are significantly influenced by APOB. Nevertheless, the significance and pathomechanisms of APOB in HCC have not been conclusively determined. We assessed APOB expression levels in HCC using three publicly available databases of TIMER2.0, UALCAN and Human Protein Atlas. To identify the biological function of APOB, we conducted enrichment analysis via LinkedOmics. Moreover, UALCAN was employed to assess the relationship between APOB expression and clinicopathological features among HCC patients. Additionally, the Kaplan–Meier plotter was utilized to investigate the prognostic relevance of APOB in HCC. To explore potential regulatory ncRNAs that could bind to APOB, we utilized StarBase and GEPIA. Furthermore, the correlation between APOB expression and immune cell infiltration, as well as immune checkpoint genes, was investigated using Spearman's correlation analysis in TISIDB, GEPIA, and TIMER2.0. The findings of our investigation showed a notable decrease in the expression levels of APOB among individuals diagnosed with HCC. Moreover, a noteworthy correlation was observed between the expression of APOB and immune checkpoint genes, alongside the occurrence of immune cell infiltration. The levels of APOB expression in HCC tissues also showed correlations with various clinicopathological features. According to Cox regression analysis, decreased APOB expression emerged as a potential autonomous predictor for OS, RFS, DSS, and PFS among HCC patients. Furthermore, we identified six potential pathways associated with non-coding RNA (ncRNA) as the most promising pathway for APOB in HCC. Our results illuminate the possible involvement of APOB in HCC and offer understanding into its governing mechanisms and medical importance.

**Supplementary Information:**

The online version contains supplementary material available at 10.1007/s12672-024-00877-6.

## Introduction

Hepatocellular carcinoma (HCC) is a prevalent cancer recognized for its unfavorable outlook due to its high fatality rate and complex origins [1, 2]. It holds the second position globally in terms of deaths related to cancer [3, 4]. Despite the progress in treatment options for HCC, such as radiofrequency ablation, transplantation, transarterial chemoembolization, and surgical resection, the survival rate remains disappointingly low [5–8]. Hence, there is an urgent need to create efficient measures for HCC that can enhance the quality of life and boost the chances of survival [9–11].

In this context, immunotherapy for cancer treatment has been gaining increased attention [12–16]. Several researchers have discovered that blockers of cytotoxic T lymphocyte-associated antigen 4, programmed cell death-ligand 1, and programmed cell death-1 elicit anti-cancer effects on HCC cells [17–19]. Nonetheless, immunotherapy provides advantages solely to a small portion of individuals, underscoring the necessity for enhanced biomarkers linked to HCC [20, 21].

Among the apolipoprotein family members, APOB consists of tiny particles that carry dietary lipids through the bloodstream from the intestines to the liver [22, 23]. APOB primarily produces two isoforms of proteins, namely apoB-100 and apoB-48 [24, 25], which are predominantly present in the serum. It is worth noting that previous research has shown a connection between APOB and diverse forms of cancer, such as gallbladder cancer [26, 27], low-grade glioma [28], non-small cell lung cancer [29, 30], and primary small cell carcinoma of the esophagus [31]. Furthermore, research conducted by Lee and colleagues indicated that individuals with HCC who have a deactivated APOB gene experience worse results [32]. According to another research, it was proposed that the metabolic reprogramming of HCC could occur due to a substantial decrease in APOB caused by hypermethylation [33]. Nevertheless, the specific cause of this connection remains unknown.

Initially, we assessed the concentrations of APOB in HCC. Subsequently, we performed an analysis of clinical parameters associated with APOB and conducted survival analysis. Moreover, the investigation of APOB regulation in HCC involved the studing non-coding RNA (ncRNA), such as miRNAs and lincRNAs. Lastly, the investigation of the relationships between APOB levels and immune cell infiltration, as well as immune checkpoints, was also conducted in HCC. Our results suggest that down-regulation of APOB, facilitated by ncRNAs, correlates with adverse prognostic outcomes and the infiltration of immune cells among HCC patients.

## Materials and methods

### TIMER2.0

The TIMER2.0 tool is a comprehensive software tool (http://timer.comp-genomics.org/) that facilitates the systematic analysis of immune infiltrates across various cancer types [34–36]. It provides estimations of immune infiltrate abundances through the utilization of multiple immune deconvolution methods. This enables users to generate visually appealing and informative figures, allowing for a comprehensive exploration of genomic characteristics, clinical attributes, and tumor immunological aspects. In the context of HCC, TIMER2.0 was employed to evaluate the prevalence of tumor infiltrates and establish relationships between APOB levels and the expression of immune checkpoints, as well as the levels of immune cell infiltration.

### LinkedOmics

LinkedOmics enables the execution of multi-omics analysis on TCGA datasets (http://www.linkedomics.org/login.php) [37, 38]. The TCGA-LIHC project was selected for analysis, encompassing a cohort of 371 HCC patients (Data type: RNA-seq; Date: 01/28/2016). The genes that showed differential expression and were associated with APOB were acquired from the LinkFinder module. The Pearson correlation coefficient was utilized for evaluation, and the results were represented using heat maps and volcano plots. Additionally, gene set enrichment analysis (GSEA) was conducted using the LinkInterpreter module for Gene Ontology (GO) and Kyoto Encyclopedia of Genes and Genomes (KEGG).

### ULACAN

The UALCAN platform offers comprehensive analyses of transcriptional data derived from The Cancer Genome Atlas (TCGA) (http://ualcan.path.uab.edu/index.html) [39, 40]. This database was used to determine APOB levels correlated with various clinical and pathological parameters (sex, nodal metastasis status, and tumor grade cancer stage) of HCC. The APOB mRNA data is quantified as Transcript per million. Disparities in APOB expression between two groups were assessed through the utilization of Welch's T-test [41].

### Human protein Atlas

The Protein Atlas for Humans offers immunohistochemical data on the expression of proteins in 20 different types of cancer, with each type consisting of 12 distinct tumors (https://www.proteinatlas.org) [42, 43]. This can be utilized for the identification of proteins specific to different tumor types that exhibit differential expression. The Human Protein Atlas presents an analysis of the proteome of liver cancer, utilizing transcriptomic data from 365 patients sourced from TCGA, along with antibody-based protein data. The specific antibody employed in this investigation is CAB016070. The immunohistochemistry profiling in selected tissues reports the presence of antibody staining in the annotated cell types as either undetected, minimal, moderate, or intense. A comparison was made between the levels of APOB protein in normal and HCC tissues using an immunohistochemistry image.

### Kaplan–Meier plotter analysis

The Kaplan–Meier plotter is a comprehensive database designed for assessing the influence of miRNAs or genes on survival outcomes across different tumor types (http://kmplot.com/analysis/) [44–46]. This database was utilized to investigate the relationship between APOB and several clinic-pathological features (gender, AJCC stage T, race, grade, stage, alcohol consumption, vascular invasion, sorafenib treatment, as well as hepatitis virus) in HCC. The survival analysis for APOB expression in overall survival (OS), Disease-specific survival (DSS), relapse-free survival (RFS, also called DFS), and progression-free survival (PFS) [47] was conducted using a total of 364, 370, 316, and 362 samples, respectively. The median value was employed as the cutoff.

### StarBase

To identify potential regulators of APOB expression in HCC, we searched for miRNAs that target APOB using StarBase, a database that integrates miRNA-target interactions from various sources (http://starbase.sysu.edu.cn/) [48, 49]. We then selected three miRNAs (hsa-miR-21-5p, hsa-miR-9-5p, and hsa-miR-877-5p) that were predicted to bind to APOB in more than three cancer types. Next, we investigated the possible lincRNAs that interact with these miRNAs using StarBase. We also performed expression correlation analyses between APOB, miRNAs, and lincRNAs in HCC samples from TCGA.

### TISIDB

The objective of the TISIDB database is to predict responses to immunotherapy by forecasting the interactions between the immune system and tumor tissue (http://cis.hku.hk/TISIDB) [50, 51]. We sought to investigate the relationships between APOB expression and chemokines, as well as immunomodulators, using RNA-Seq data from a cohort of 373 patients with TCGA-LIHC obtained from the TISIDB database. Statistical significance was detected by considering P values below 0.05 and correlation coefficients exceeding 0.2 or falling below − 0.2 [52].

### GEPIA

To examine the role of lincRNAs in HCC progression and prognosis, we employed GEPIA, a web-based tool that facilitates gene expression analysis utilizing data from TCGA and GTEx data (http://gepia.cancer-pku.cn/index.html) [53, 54]. The expression levels of lincRNAs were compared between HCC tumors and normal liver tissues. Additionally, survival analyses for lincRNAs among HCC patients were conducted using Kaplan–Meier plots.

## Results

### APOB levels are decreased in HCC patients

**Fig. 1:**
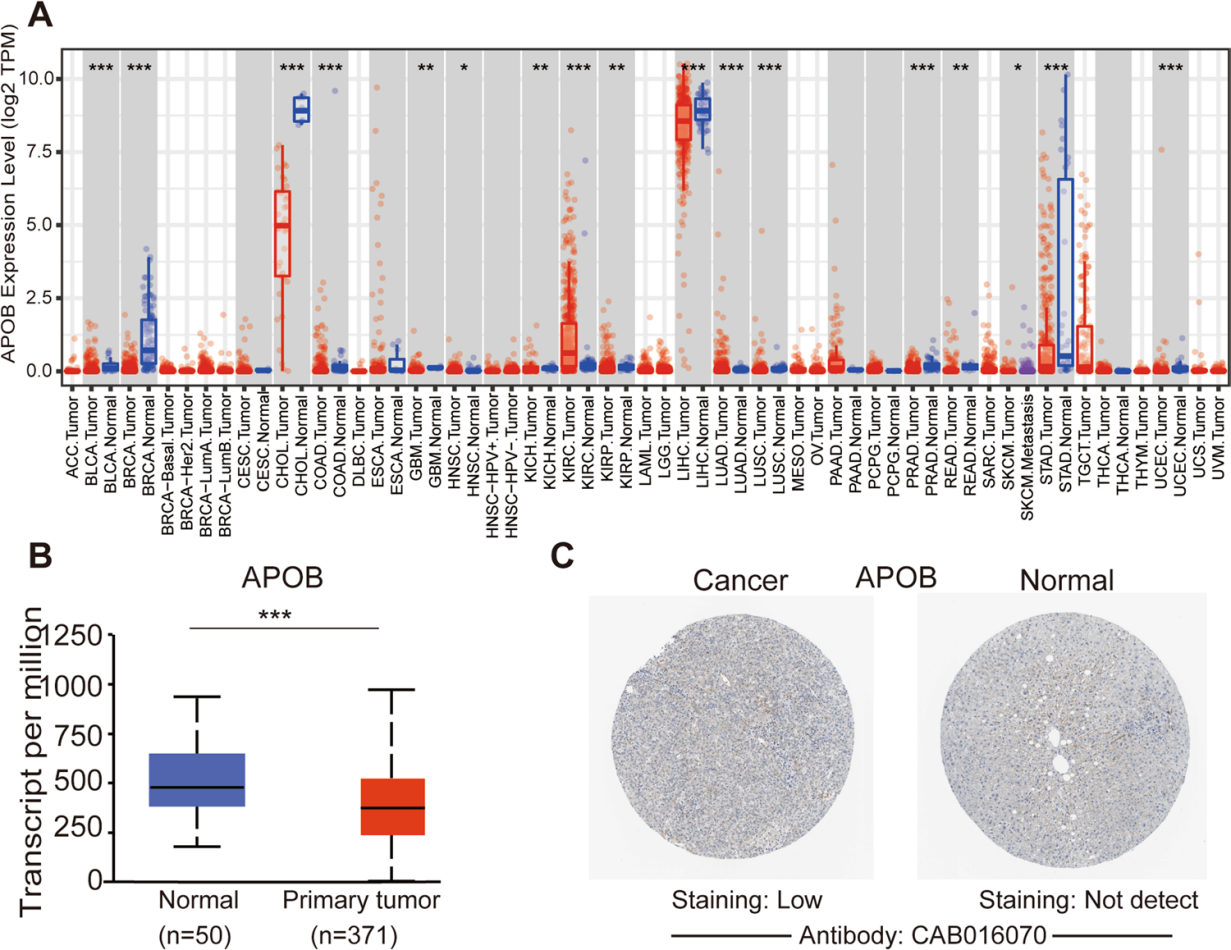
Expression of APOB in HCC. **A** TIMER was used to examine APOB expression in a variety of cancer types (**B**) mRNA expression of APOB in HCC tissues and adjacent normal liver tissues (UALCAN). **C** immunohistochemical images of APOB in HCC tissues and normal liver tissues (Human Protein Atlas). Note: * p < 0.05, ** p < 0.01,*** p < 0.001

Initially, the TIMER 2.0 was employed to investigate the level of APOB mRNA in human tumors. Figure 1A showed a decrease in APOB levels in cholangiocarcinoma (CHOL), liver HCC (LIHC), and breast invasive carcinoma (BRCA) compared to normal tissues. Moreover, analysis of the UALCAN databases revealed a substantial decrease in APOB mRNA expression in HCC patients relative to normal tissues (Fig. 1B).

Next, the Human Protein Atlas was utilized to assess the protein levels of APOB in HCC. In normal liver tissues, APOB protein was undetectable, as depicted in Fig. 1C, while HCC tissues exhibited detectable but minimal levels.

### Enrichment analyses of APOB Co-expressed Genes in HCC

**Fig. 2:**
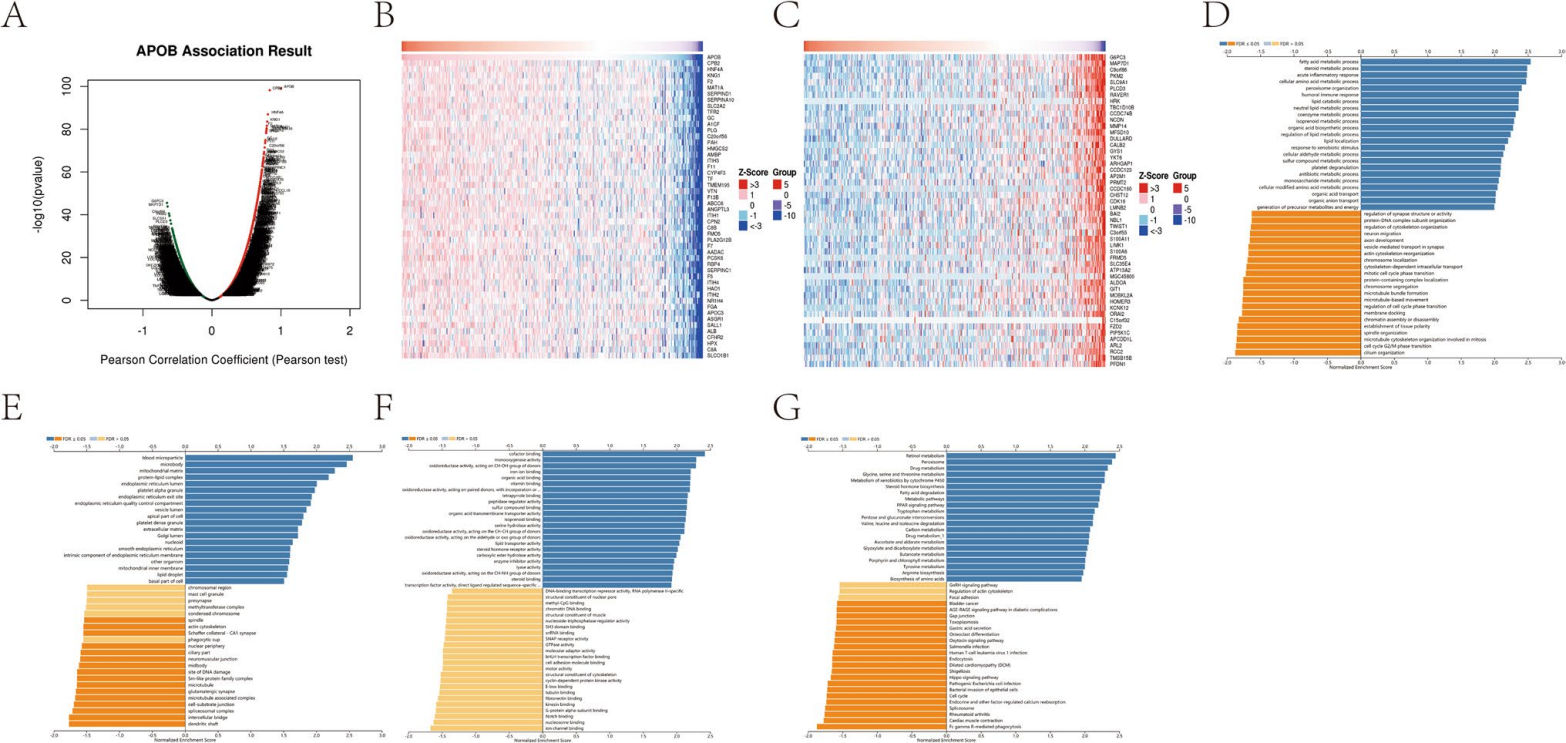
Enrichment analyses of APOB co-expressed genes. APOB co-expressed genes was display by volcano map (**A**), Heat map of top 50 positively (**B**) and 50 negatively (**C**). GO enrichment analysis for biological processes (**D**), cellular components (**E**), molecular functions (**F**) and KEGG (**G**)

To explore the biological functions and pathways of APOB in HCC, there was a positive correlation between APOB and 3843 genes, whereas APOB exhibited a negative correlation with 8125 genes (Fig. 2A). The heat maps illustrating the top 50 genes associated with APOB are presented in Fig. 2B and Fig. 2C. The examination of biological processes (BP) revealed that APOB's co-expressed genes were significantly concentrated in the metabolic process of fatty acids, immediate inflammatory reaction, reaction to foreign substances, control of synapse structure or activity, and control of cytoskeleton organization (Fig. 2D). The analysis of cellular components (CC) revealed that APOB's co-expressed genes were notably clustered in blood microparticle, microbody, dendritic shaft, actin cytoskeleton, and endoplasmic reticulum lumen (Fig. 2E). The analysis of molecular functions (MF) revealed that co-expressed genes showed significant enrichment in molecular adaptor activity, binding to cell adhesion molecules, binding to snRNA, binding to cofactors, and binding to organic acids (Fig. 2F). Based on the examination of KEGG, the co-expressed genes were primarily concentrated in drug processing, pathways related to fatty acids, carbon processing, the cell cycle, and the control of actin cytoskeleton (Fig. 2G).

### APOB levels and clinical characteristics for HCC patients

**Fig. 3:**
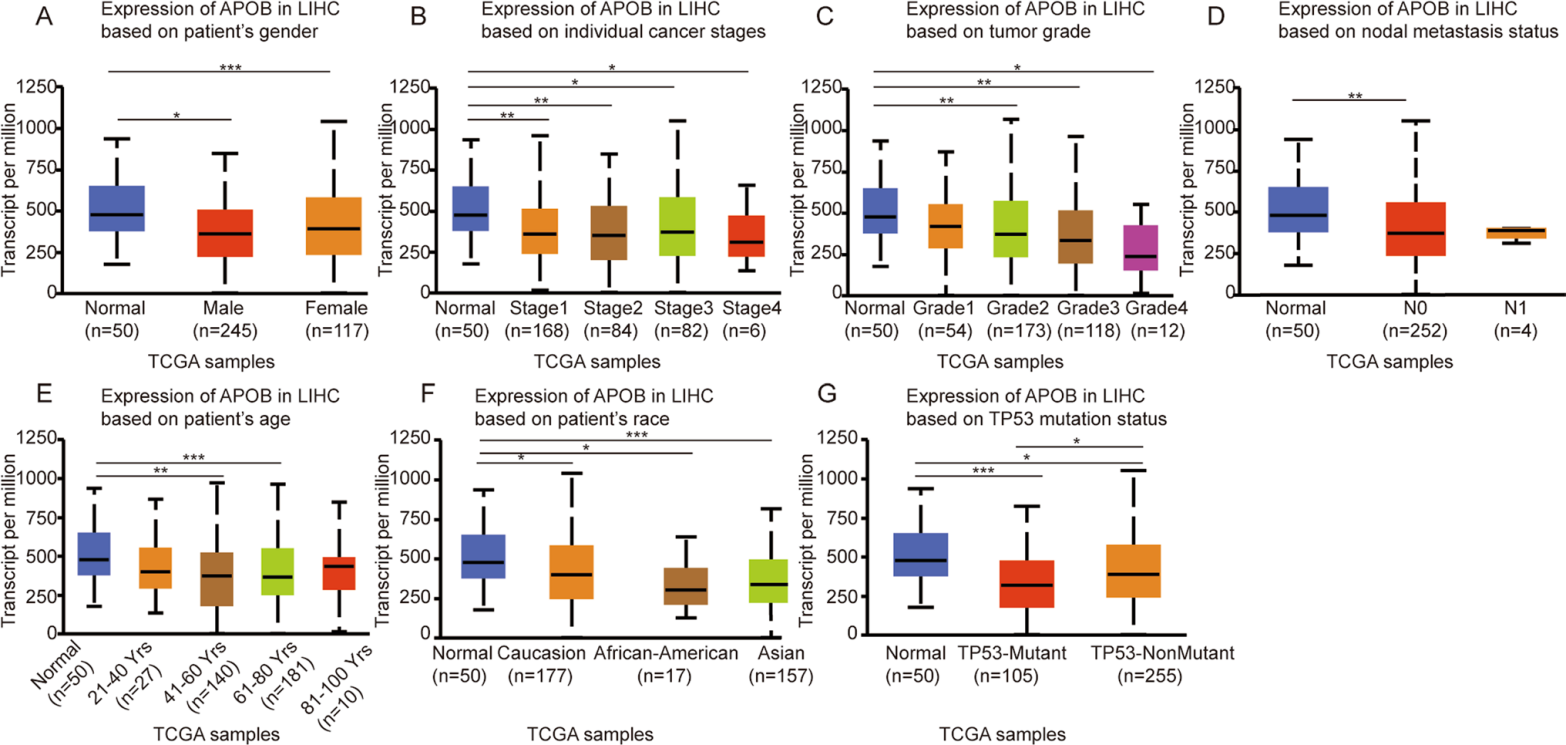
The UALCAN database was used to quantify APOB expression based on clinical parameters among patients in different groups. **A**–**D** Analysis was shown for gender (**A**), cancer stages (**B**), tumor grade (**C**), metastasis (**D**), age (**E**), race (**F**) and TP53 mutation status (**G**). Differences of APOB expression between two groups were compared using Welch's T-test: * p < 0.05, ** p < 0.01,*** p < 0.001

Through the UALCAN online tool, we evaluated APOB levels among various patient groups based on several parameters. Compared with normal controls, APOB levels were markedly decreased in both females and males with HCC (Fig. 3A). With regards to cancer stage, APOB levels were lower in HCC patients classified as stages 1,2, 3 and 4 (Fig. 3B). Based on tumor stage, a marked decrease in APOB levels was detected in HCC patients in grades 2, 3 and 4 (Fig. 3C). As for nodal metastasis, APOB levels were lower in HCC patients classified as N0 (Fig. 3D). Regarding age, there was a significant decline in APOB levels in HCC patients in aged 41–60 years and 61–80 years (Fig. 3E). APOB expression was dramatically decreased in HCC patients of african-american, caucasian and asian origin (Fig. 3F). In addition, Down-regulation of APOB levels were observed in both TP53 wild-type and TP53-mutant in HCC patients (Fig. 3G).

### Prognostic significance of APOB Levels in HCC

**Fig. 4:**
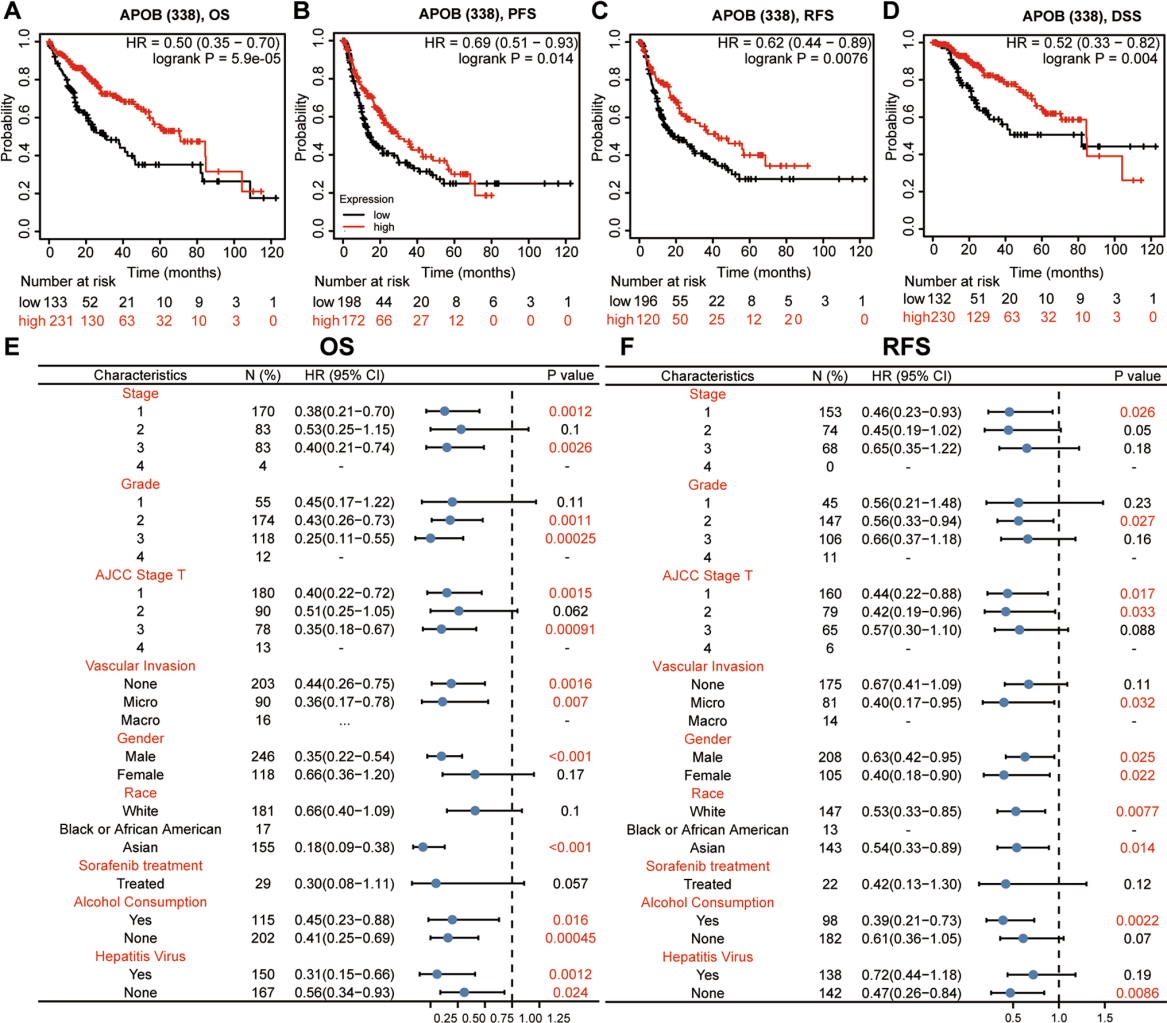
APOB's prognostic value was assessed by the Kaplan–Meier plot. **A**–**D** The Kaplan–Meier plot is shown for OS (**A**), PFS (**B**), RFS (**C**) and DSS (**D**).A forest plot shows the correlation between APOB expression and clinicopathological parameters in HCC patients (Kaplan–Meier Plotter) (**E**–**F**)

The Kaplan–Meier plotter was employed toinvestigatethe prognostic significance of APOB expression amongHCCpatients. Statistically significant findings were observed for the four survival outcomes (Fig. 4A–D). Subsequently, a Cox proportional hazards model was used to estimate hazard rates with a 95% confidence interval. The findings indicated that decreased APOB levels in patients with HCC were linked to unfavorable prognostic results, such as OS (HR = 0.50, 95% CI 0.35–0.70, P = 5.9e-5; Fig. 4A), PFS (HR = 0.69, 95% CI 0.51–0.93, P = 0.014; Fig. 4B), RFS (HR = 0.62, 95% CI 0.44–0.89, P = 0.0076; Fig. 4C), DSS (HR = 0.52, 95% CI 0.33–0.82, P = 0.004; Fig. 4D). Moreover, we conducted the cox proportional hazards model to investigate the relationship between APOB levels and various clinical features in HCC (as shown in Fig. 4E and F). Our observation revealed a correlation between decreased APOB levels and unfavorable OS and RFS in HCC patients, irrespective of tumor grade, stage, gender, vascular invasion, AJCC stage T, race, alcohol consumption, and hepatitis virus.

### Analysis and prediction of miRNAs associated with APOB

**Fig. 5:**
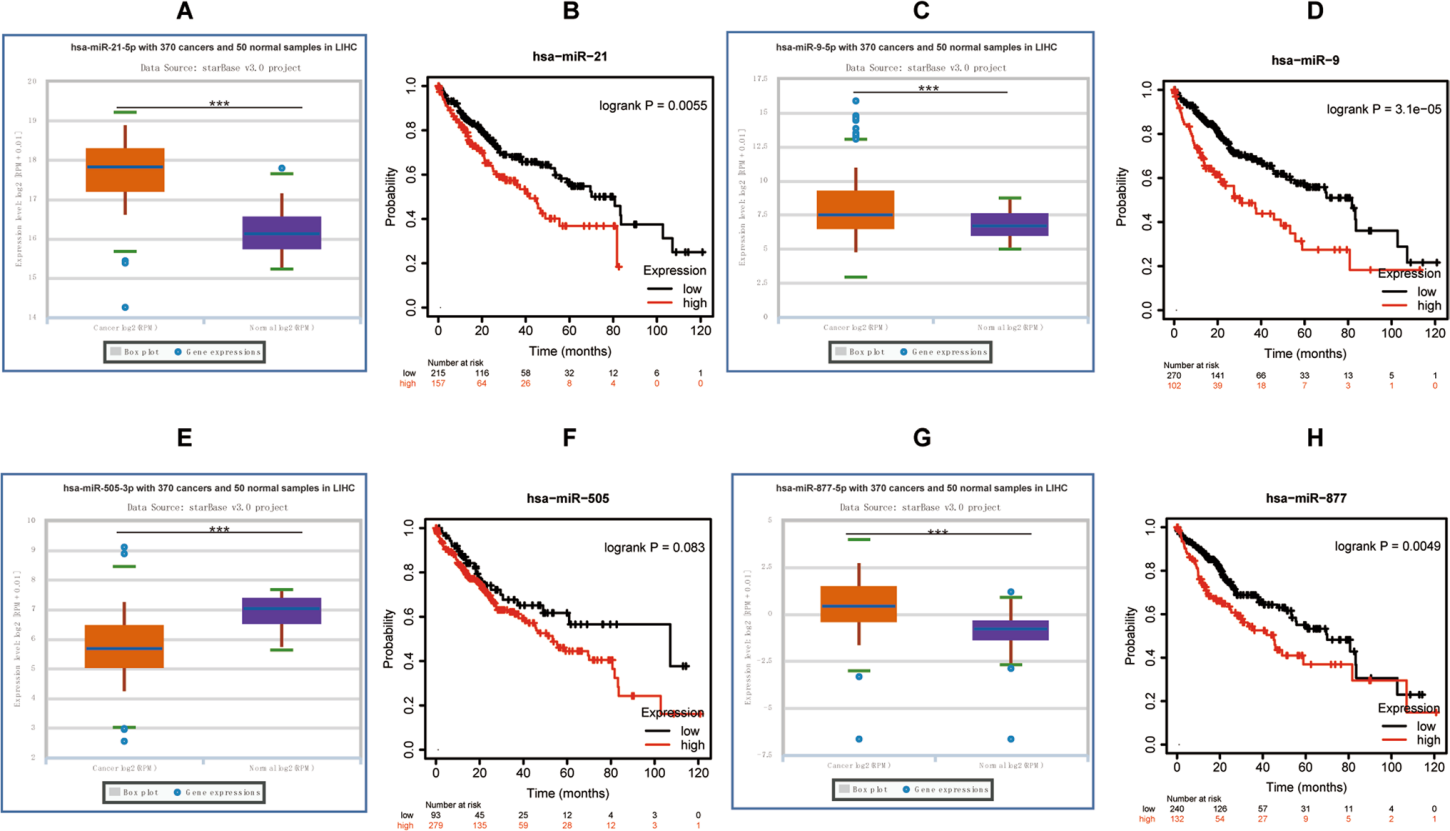
Identification of potential miRNAs of APOB and their prognostic value in HCC. (A/C/E/G) The expression of has-miR-21-5p (**A**), has-miR-9-5p (**C**), hsa-miR-505-3p (**E**) and hsa-miR-877-5p (**G**) in HCC. (**B**/**D**/**F**/**H**) The OS analysis for has-miR-21-5p (**B**), has-miR-9-5p (**D**), hsa-miR-505-3p (**F**) and hsa-miR-877-5p (**H**) in HCC. Differences of four miRNAs expression between two groups were compared using hypergeometric tests: * p < 0.05, ** p < 0.01,*** p < 0.001

NcRNAs are vital in gene expression regulation. To investigate the potential influence of various ncRNAs on APOB, we employed the starbase database to predict miRNAs that could potentially interact with APOB. As a result, six miRNAs were identified (Table 1). In the context of HCC, APOB exhibited a significant inverse correlation with hsa-miR-21-5p, hsa-miR-9-5p, and hsa-miR-877-5p while displaying a favorable correlation with hsa-miR-505-3p. Subsequently, we conducted a comprehensive analysis to assess the expression levels and prognostic relevance of these four miRNAs in HCC. The expression of Hsa-miR-21-5p, hsa-miR-9-5p, and hsa-miR-877-5p was significantly increased in HCCs, and reducing their levels was linked to favorable patient outcomes (Fig. 5).

**Table 1:** The expression correlation between predicted miRNAs and APOB in HCC analyzed by starBase database

Gene	miRNA	R-value	P-value
APOB	hsa-miR-15a-5p	− 0.060	0.2530
APOB	hsa-miR-21-5p	− 0.156	**0.0026**
APOB	hsa-miR-9-5p	− 0.111	**0.0331**
APOB	hsa-miR-505-3p	0.111	**0.0331**
APOB	hsa-miR-629-5p	− 0.038	0.4620
APOB	hsa-miR-877-5p	− 0.134	**0.0100**

### Prediction and analysis of related LincRNAs of Hsa-miR-21-5p, Hsa-miR-9-5p, and Hsa-miR-877-5p

**Fig. 6:**
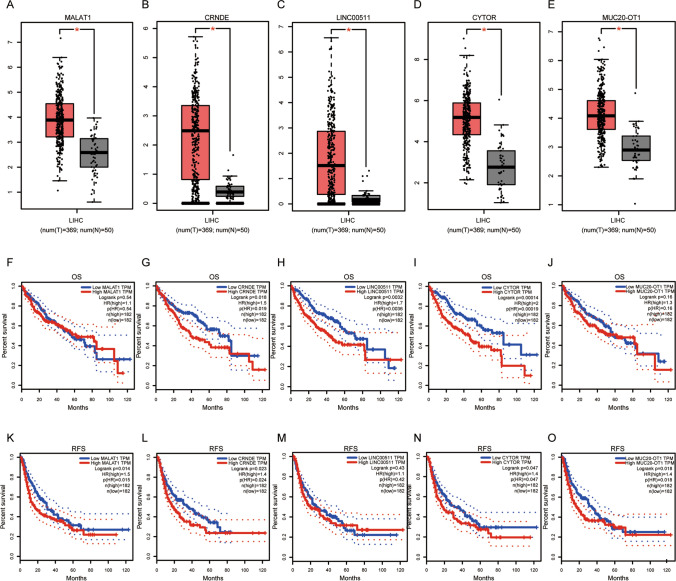
Expression analysis and survival analysis for related lincRNAs of hsa-miR-21-5p, hsa-miR-9-5p and hsa-miR-877-5p in HCC. **A**–**E** The expression of MALAT1 (**A**), CRNDE (**B**), LINC00511 (**C**), CYTOR (**D**) and MUC20-OT1 (**E**) in TCGA HCC compared with TCGA normal data. (**F**–**J**) The OS analysis for MALAT1 (**F**), CRNDE (**G**), LINC00511 (**H**), CYTOR (**I**) and MUC20-OT1 (**J**). **K**–**O** The RFS for MALAT1 (**K**), CRNDE (**L**), LINC00511 (**M**), CYTOR (**N**) and MUC20-OT1 (**O**) in HCC. Differences of lincRNAs expression between two groups were compared using one-way ANOVA: * p < 0.05, ** p < 0.01,*** p < 0.001

According to studies, long intergenic non-coding RNA (lincRNA) can bind to miRNA, similar to mRNA, and subsequentlyregulate mRNA expression directly [55, 56]. Next, to construct a regulatory network of mRNA-miRNA-lincRNA, we utilized starbase database to explore related lincRNA associated with hsa-miR-21-5p/hsa-miR-9-5p/hsa-miR-877-5p. A total of 18, 24 and 35 possible lincRNAs were forecasted for hsa-miR-21-5p, hsa-miR-9-5p and hsa-miR-877-5p, respectively. In order to enhance the graphical representation, a regulatory network for these microRNAs was constructed using cytoscape software (Supplementary Fig. 1 and Supplementary Table 1–Supplementary Table 3). Subsequently, the expression levels of these lincRNAs were evaluated in HCC patients utilizing the GEPIA platform. Figure 6A–E and Supplementary Fig. 2A-2D showed that among all the related lincRNAs, only MALAT1, CRNDE, LINC00511, CYTOR, MUC20-OT1, LINC01089, PTV1, LINC00665 and FLVCR1-AS1 were markedly elevated in HCC, relative to normal controls. Furthermore, prognostic values of these lincRNAs were determined in HCC. As revealed in Fig. 6F–O and Supplementary Fig. 2E-2L, only HCC patients with elevated levels of CRNDE and CYTOR exhibited poorer OS and RFS. In addition, overexpression of LINC00511 implied poor OS for HCC patients, whereas high expression of MALAT1, MUC20-OT1, LINC01089 showed poor RFS.

The relationship between these six lincRNAs and hsa-miR-21-5p, hsa-miR-9-5p, and hsa-miR-877-5p, as well as APOB, in HCC patients was further investigated using the starbase database. In Table 2, lincRNA correlated positively with miRNA, whereas mRNA correlated negatively with lincRNA. Based on expression, survival, and correlation analyses, MALAT1, CRNDE, LINC00511, CYTOR, MUC20-OT1 and LINC01089 might be the six most potential related lincRNAs of hsa-miR-21-5p, hsa-miR-9-5p, and hsa-miR-877-5p in HCC. A total of six axes were found in HCC (MALAT1/hsa-miR-21-5p/APOB; CRNDE/hsa-miR-9-5p/APOB;LINC00511/hsa-miR-9-5p/APOB;CYTOR/hsa-miR-877-5p/APOB;MUC20-OT1/hsa-miR-877-5p/APOB; LINC01089/hsa-miR-877-5p/APOB).

**Table 2:** Correlation analysis between lincRNA and hsa-miR-21-5p/hsa-miR-9-5p/hsa-miR-877-5p or lincRNA and APOB in HCC determined by starBase database

lincRNA	miRNA	R value	P value
MALAT1	hsa-miR-21-5p	-0.06	0.253
CRNDE	hsa-miR-9-5p	0.145	0.00535
LINC00511	hsa-miR-9-5p	0.231	< 0.001
CYTOR	hsa-miR-877-5p	0.283	< 0.001
MUC20-OT1	hsa-miR-877-5p	0.113	0.0291
LINC01089	hsa-miR-877-5p	0.188	< 0.001

MALAT1	APOB	− 0.156	0.00253
CRNDE	APOB	− 0.172	0.00084
LINC00511	APOB	− 0.239	< 0.001
CYTOR	APOB	− 0.380	< 0.001
MUC20-OT1	APOB	− 0.125	0.0157
LINC01089	APOB	− 0.252	< 0.001

### Correlation between APOB expression and the extent of immune cell infiltration in HCC

**Fig. 7:**
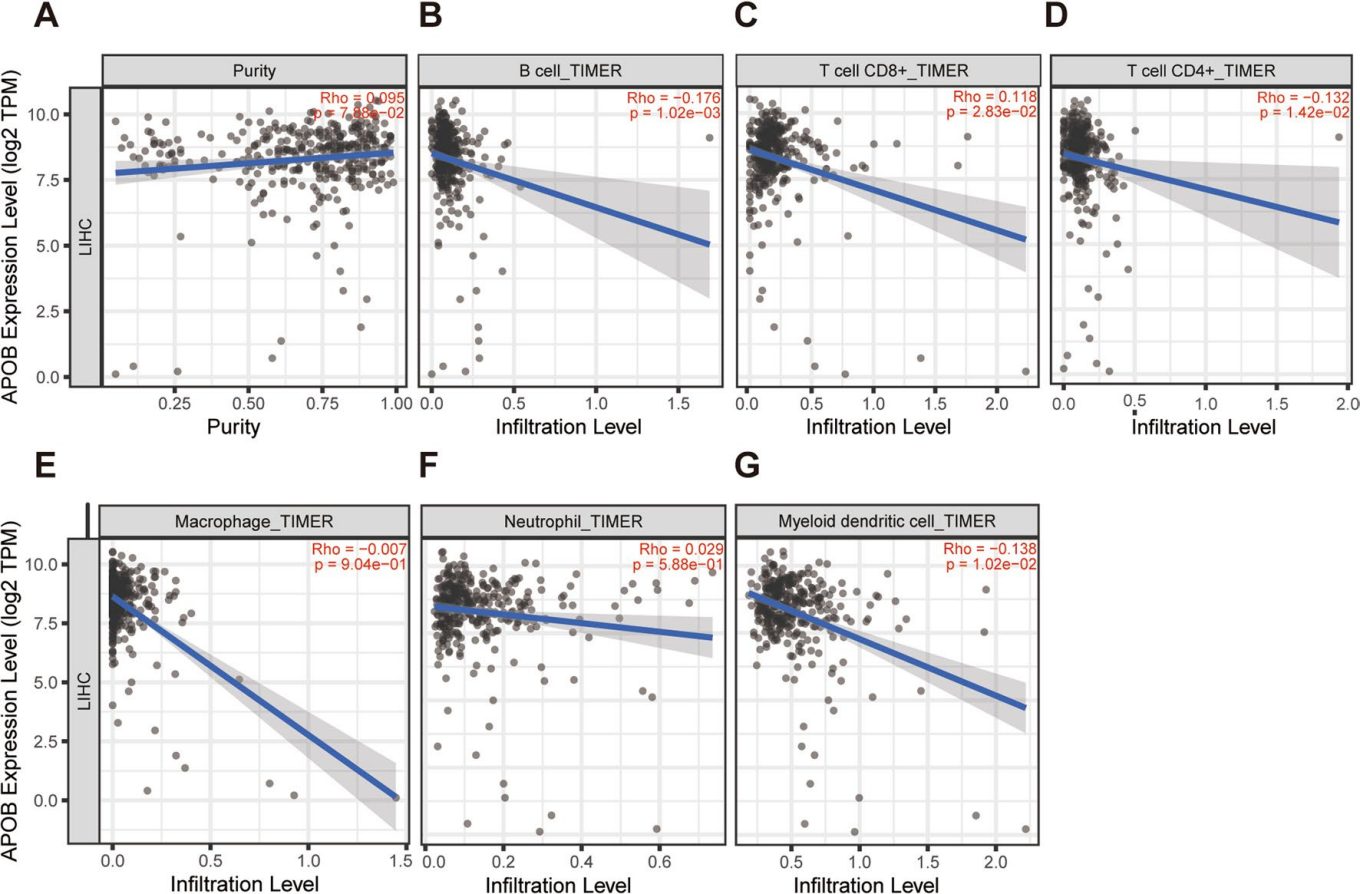
Immune cell infiltration in HCC was correlated with APOB expression level (**A**–**G**). The correlation of APOB expression level with purity (**A**), B cell (**B**), CD8 + T cell (**C**), CD4 + T cell (**D**), macrophage (**E**), neutrophil (**F**), dendritic cell (**G**) infiltration level in HCC

**Fig. 8:**
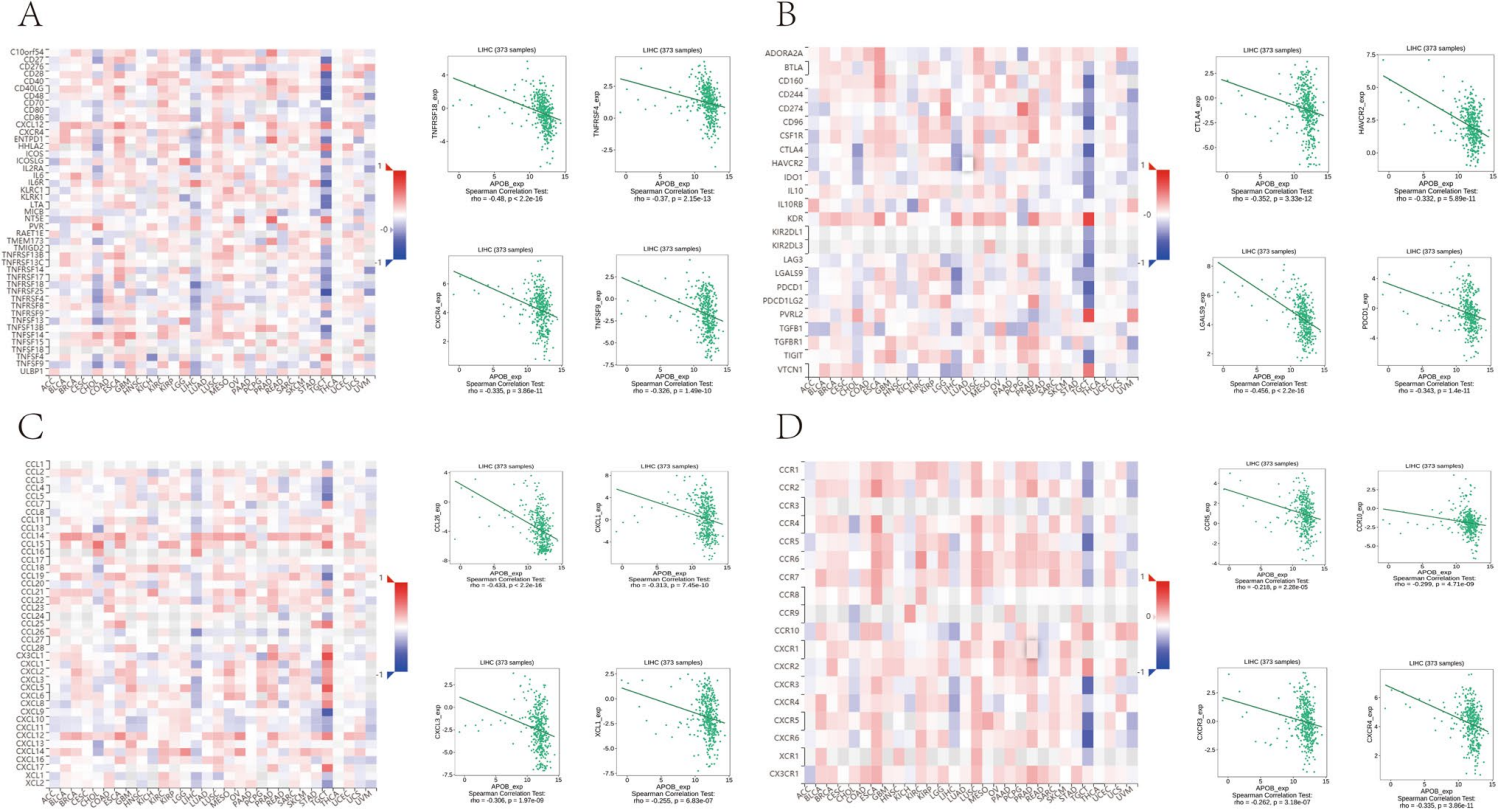
The relationship between APOB expression and immunostimulators (**A**), immunoinhibitors (**B**), chemokines (**C**) and chemokine receptors (**D**)

In TIMER 2.0, the Spearman correlation coefficient was employed to assess the relationship between APOB levels and levels of immune cell infiltration. In the context of HCC, Fig. 7B–G presented compelling evidence of an inverse correlation between APOB levels and the presence of CD4 + T cells, dendritic cells, and B cells, while APOB levels displayed a positive correlation with CD8 + T cells. Furthermore, an exploration of the TISIDB database allowed for an examination of the connections between APOB expression and the infiltration of immune cells. A significant association was discovered between APOB and immune boosters, like CXCR4 (*r* = − 0.335 and *p* = 3.86e-11), TNFRSF4 (*r* = − 0.37 and *p* = 2.15e-13), TNFSF9 (*r* = − 0.326 and *p* = 1.49e-10), and TNFRSF18 (*r* = − 0.48 and *p* < 2.2e-16) (Fig. 8A). Moreover, APOB exhibited a strong correlation with immune suppressors, such as CTLA4 (*r* = − 0.352 and *p* = 3.33e-12), HAVCR2 (*r* = − 0.332 and *p* = 5.89e-11), PDCD1 (*r* = − 0.343 and *p* = 1.4e-11), and LGALS9 (*r* = -0.456 and *p* < 2.2e-16) (Fig. 8B). Furthermore, a notable correlation was observed between APOB and chemokine, including CXCL1 (*r* = -0.313 and *p* = 7.45e-10), CCL26 (*r* = − 0.433 and *p* < 2.2e-16), CXCL3 (*r* = − 0.306 and *p* = 1.97e-09), and XCL1 (*r* = − 0.255 and *p* = 6.83e-07) in Fig. 8C. Finally, there was a strong association between APOB and chemokine receptors, specifically CCR10 (*r* = − 0.299 and *p* = 4.71e-09), CCR5 (*r* = − 0.218 and *p* = 2.28e-05), CXCR3 (*r* = − 0.262 and *p* = 3.18e-07), and CXCR4 (*r* = − 0.335 and *p* = 3.86e-11) as depicted in Fig. 8D.

### Correlation between APOB and immune checkpoints expressions in HCC

**Fig. 9:**
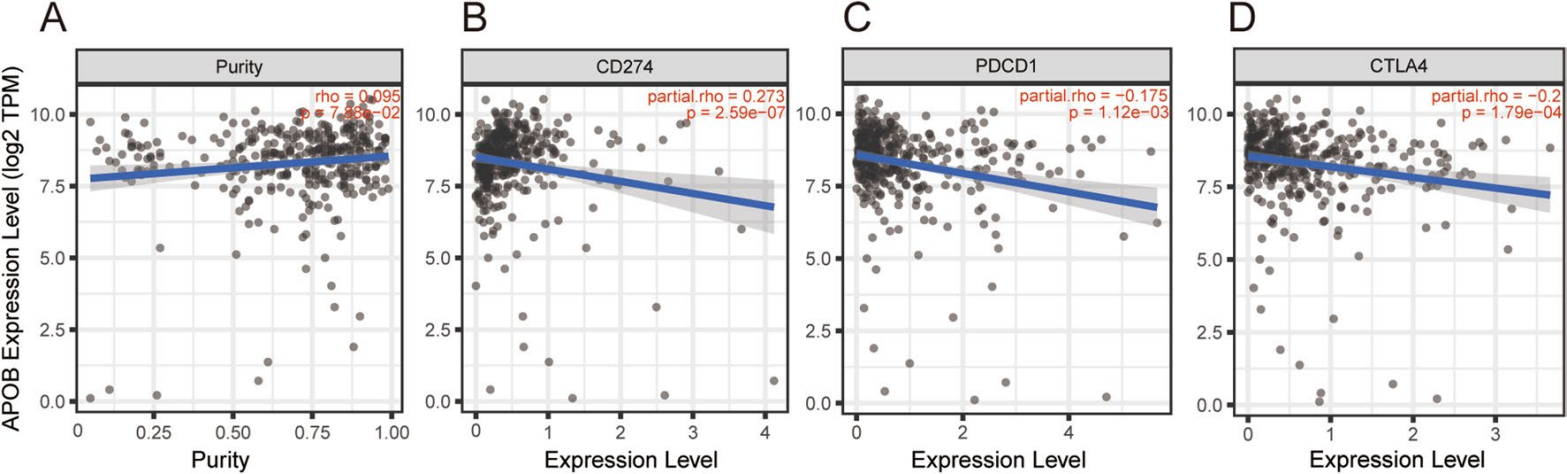
Correlation of APOB expression with CD274, PDCD1, and CTLA4 expression in HCC. **A**–**D** Spearman correlation of APOB with expression of purity (**A**), CD274 (**B**), PDCD1 (**C**), CTLA-4 (**D**) in HCC adjusted by purity using TIMER

The immune escape of tumors is significantly influenced by immune checkpoints such as CTLA4, PDCD1 and CD274. To assess the potential tumor suppressor role of APOB in HCC, we employed the Spearman correlation coefficient to evaluate the relationship between APOB and CD274, PDCD1, and CTLA4. After adjusting for purity, a significant positive correlation was observed between APOB expression and CD274 in patients with HCC, as depicted in Fig. 9B–D. Conversely, a negative association was observed between APOB expression and PDCD1 as well as CTLA4. In HCC patients, we found significant inverse associations between APOB and PDCD1 as well as CTLA4, similar to the findings from GEPIA data analysis (Supplementary Fig. 3B–C).

## Dicussion

At present, HCC is strongly linked to unfavorable prognostic results. Discovering the molecular mechanisms that cause HCC carcinogenesis could potentially enhance the development of therapeutic targets and identify valuable prognostic biomarkers. APOB plays a major role in HCC. Nevertheless, APOB’s significance in HCC should be further explored.

The expression of APOB in HCC from the TCGA project was assessed. APOB levels were verified using the UALCAN and TIMER 2.0 databases. Afterwards, we investigated the co-expressed genes of APOB and conducted enrichment analyses using LinkedOmics. The survival analysis for APOB demonstrated that HCC individuals exhibiting reduced APOB levels experienced unfavorable prognostic results. The authors Lee and colleagues [32] conducted a study of 30 individuals and revealed that patients diagnosed with HCC and with deactivated APOB experienced worse results. These results show the inhibitive role of APOB in HCC.

Currently, the exact mechanism behind the correlation between APOB and HCC is still unidentified. However, individuals diagnosed with familial hypobetalipoproteinemia (FHBL) have previously demonstrated the presence of APOB deleterious mutations, which are linked to reducedlevels of low-density lipoprotein cholesteroland overall cholesterol [57]. Individuals with FHBL caused by APOB mutations will experience the development of liver cirrhosis, hepatocarcinoma, and hepatic steatosis [58].

NcRNAs, such as microRNAs, lincRNAs, and circular RNAs (circRNAs), exert influence on gene expression by engaging in collective interactions through the ceRNA mechanism [59–61]. Using starbase, we predicted the miRNAs that could bind to APOB, ultimately identifying six regulatory miRNAs associated with APOB. The majority of these miRNAs are implicated in tumorigenesisin HCC. For example, individuals diagnosed with HCC exhibit notably increased concentrations of hsa-miR-21-5p in their blood [62]. Additionally, miR-9-5p facilitates HCC growth, movement, and infiltration by specifically targeting ESR1 [63]. Hsa-miR-505-3p is up-regulated in side population cells of HCC [64]. Moreover, the expression of hsa-miR-877-5p in HCC tissues exceeds that observed in healthy liver tissues [65]. The most potential miRNA associated with tumor progression of APOB was identified through correlation, expression, and survival analyses, specifically hsa-miR-21-5p, hsa-miR-9-5p, and hsa-miR-877-5p.

Subsequently, the potential lincRNAs associated withhsa-miR-877-5p/APOB, hsa-miR-9-5p/APOB and, hsa-miR-21-5p/APOB axes were explored in HCC. Next, related lincRNAs for hsa-miR-877-5p/APOB, hsa-miR-9-5p/APOB and hsa-miR-21-5p/APOB axes were detected. 18, 24 and 35 possible lincRNAs were found, respectively. Six of the most potential related lincRNAs, such as MALAT1, CRNDE, LINC00511, CYTOR, MUC20-OT1 and LINC01089 were identified through expression, survival and correlation analyses. Considerable research has been conducted on these six lincRNAs, revealing their roles as oncogenes in HCC. For example, MALAT1 plays a role in HCC development through upregulation of SRSF1 and activation of mTOR [66], as well as the activation of the ERK/MAPK signaling pathway, which in turn regulates metastasis-associated genes and contributes to the aggressive characteristics of HCC cells [67]. CRNDE promotes HCC proliferation, invasion and migration by regulating the miR-203/BCAT1 axis. It also enhances proliferation, migration, and chemoresistance in HCC by suppressing epigenetic factors CELF2 and LATS2 [68, 69]. LINC00511 enhances cell malignant behavior and modulates the miR-195/EYA1 axis in HCC patients [70] and regulates invadopodia formation and exosome release in HCC [71]. CYTOR enhances cell proliferation and tumor growth through the miR-125b/SEMA4C axis in HCC [72]. Taken together, the potential regulatory pathways in HCC patients were identified as the CYTOR/hsa-miR-877-5p/APOB; CRNDE/hsa-miR-9-5p/APOB; MUC20-OT1/hsa-miR-877-5p/APOB; LINC00511/hsa-miR-9-5p/APOB; LINC01089/hsa-miR-877-5p/APOB; MALAT1/hsa-miR-21-5p/APOB axis.

Radiotherapy, chemotherapy, and immunotherapy can be affected by tumor immune cell infiltrations, impacting prognostic outcomes for cancer patients [73–75]. Our findings demonstrated that APOB is markedly negatively associated with B cells, dendritic cells and CD4 + T cells in HCC. These findings suggest that tumor immune infiltrations could contribute to the inhibitory effect of APOB on HCC.

Furthermore, our study highlights the importance of immune checkpoint expression and adequate immune cell infiltration in enhancing the effectiveness of immunotherapy [76–80]. Therefore, we assessed the association between APOB and immune checkpoints. Our analysis showed a significant negative association between APOB levels and the expression levels of PDCD1 and CTLA4, implying that targeting APOB might enhance the immunotherapeutic efficacy in HCC.

In conclusion, a detrimental association exists between diminished APOB expression and an unfavorable prognosis in HCC. Six related regulatory mechanisms were identified for APOB in HCC. Additionally, APOB could suppress tumor immune cells through the reduction of immune checkpoint infiltration and expression. Nonetheless, it is imperative that future endeavors encompass additional fundamental experiments and extensive clinical trials to substantiate these findings.

### Supplementary Information

Additional file1 (DOCX 4992 KB)

Additional file2 (DOCX 20 KB)

## Data Availability

The datasets produced and/or analysed in the present study can be obtained from the corresponding author upon reasonable request.
