# Supplementary material for: APOB is a potential prognostic biomarker in hepatocellular carcinoma
Source: Discov Oncol. 2024 Feb 3;15:28. doi: 10.1007/s12672-024-00877-6 (PMC10838261; doi:10.1007/s12672-024-00877-6)
Supplement: Supplementary file 1 — Additional file1 (DOCX 4992 KB) [file 12672_2024_877_MOESM1_ESM.docx]

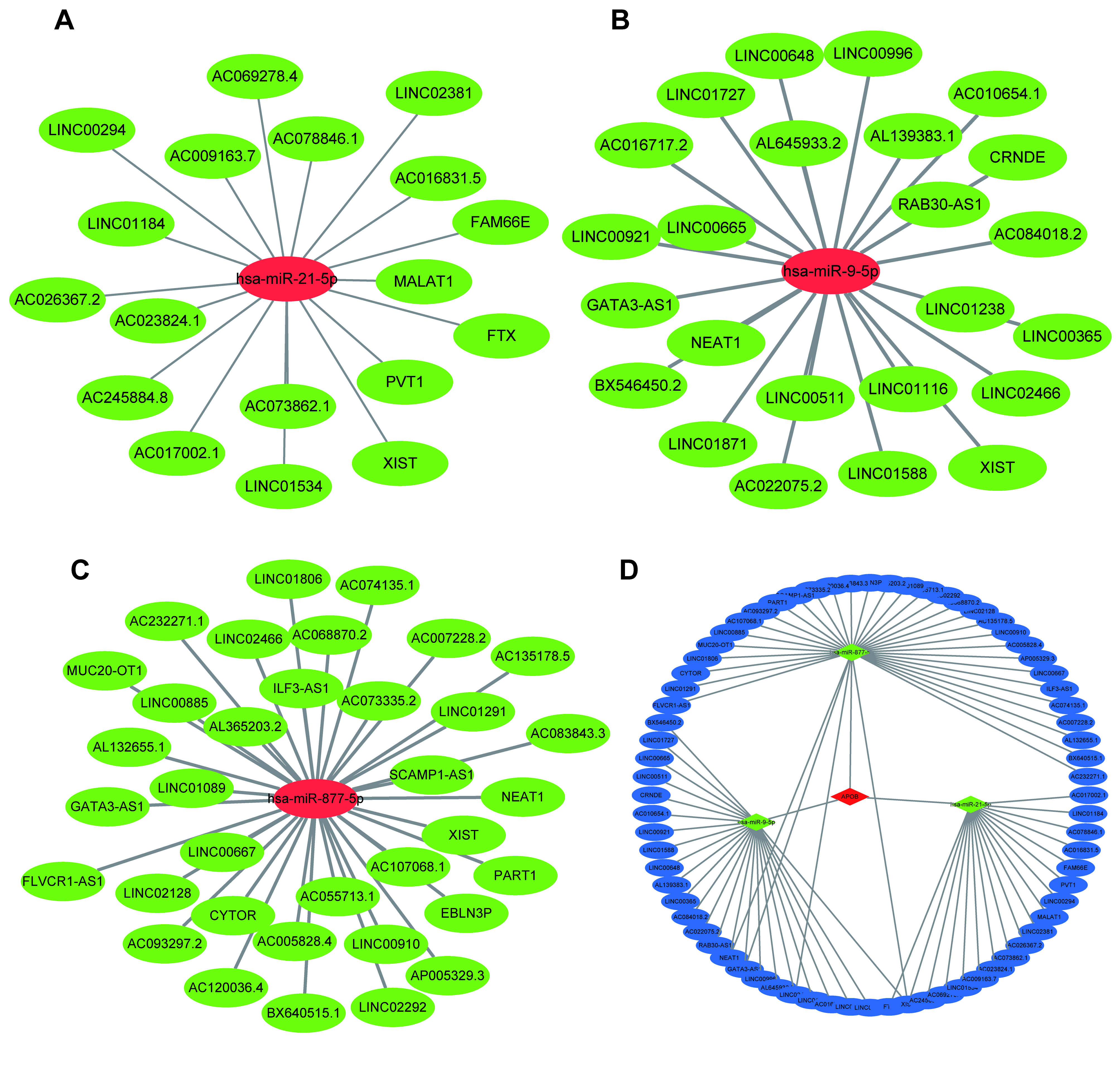


Supplementary Figure 1 The lincRNA-miRNA and ceRNA regulatory network established by cytoscape software. (A) lncRNA-hsa-miR-21-5p, (B) lncRNA-hsa-miR-9-5p, (C) lncRNA-hsa-miR-877-5p, (D) APOB-miRNA-lncRNA.


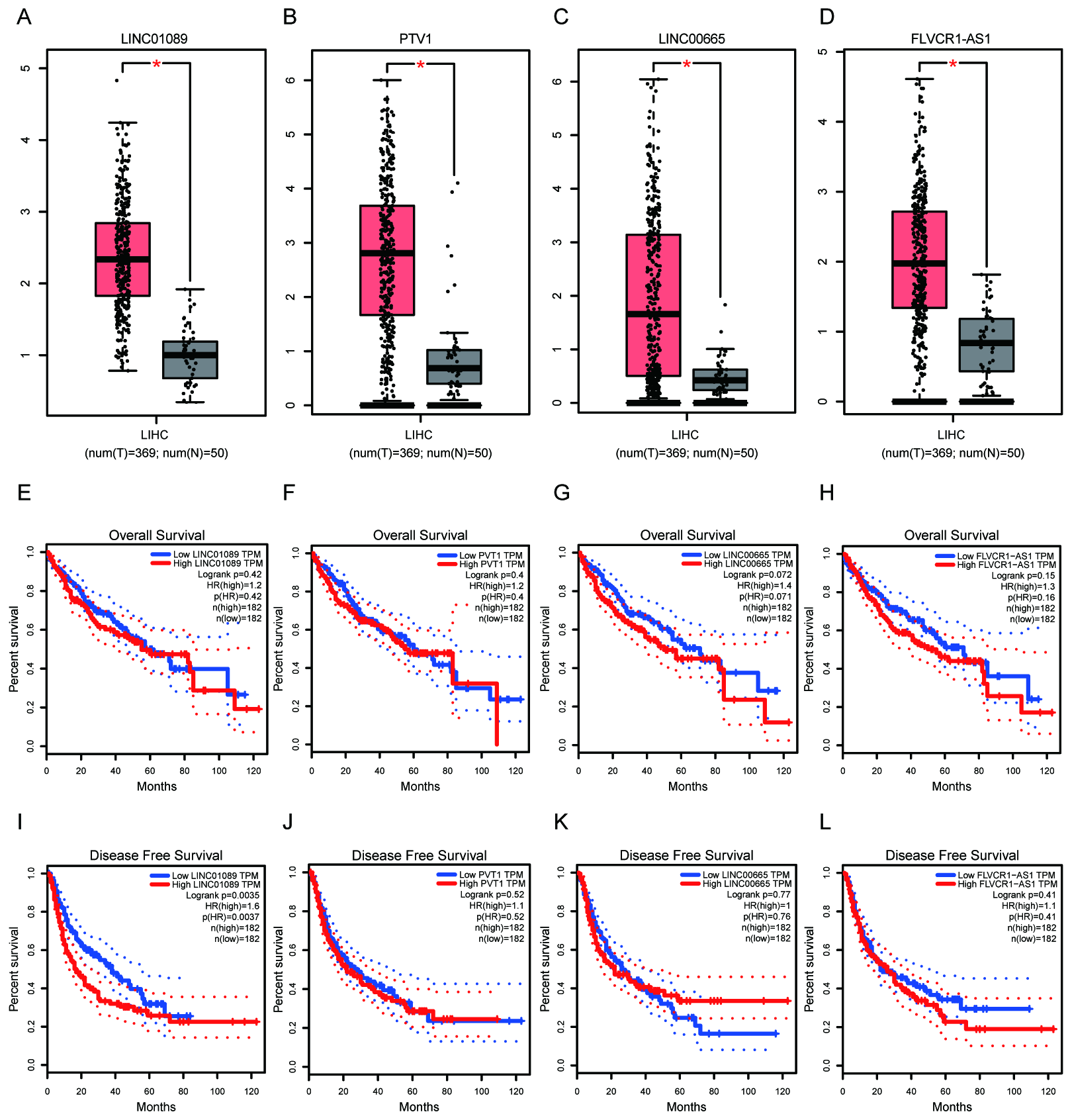


Supplementary Figure 2 Expression analysis and survival analysis for related lincRNAs of hsa-miR-21-5p, hsa-miR-9-5p and hsa-miR-877-5p in HCC. (A-D) The expression of MUC20-OT1 (A), PTV1 (B), LINC00665 (C), FLVCR1-AS1 (D) in TCGA HCC compared with TCGA normal data. (E-H) The OS analysis for MUC20-OT1 (E), PTV1 (F), LINC00665 (G), FLVCR1-AS1 (H). (I-L) The RFS for MUC20-OT1 (I), PTV1 (J), LINC00665 (K), FLVCR1-AS1 (L) in HCC.


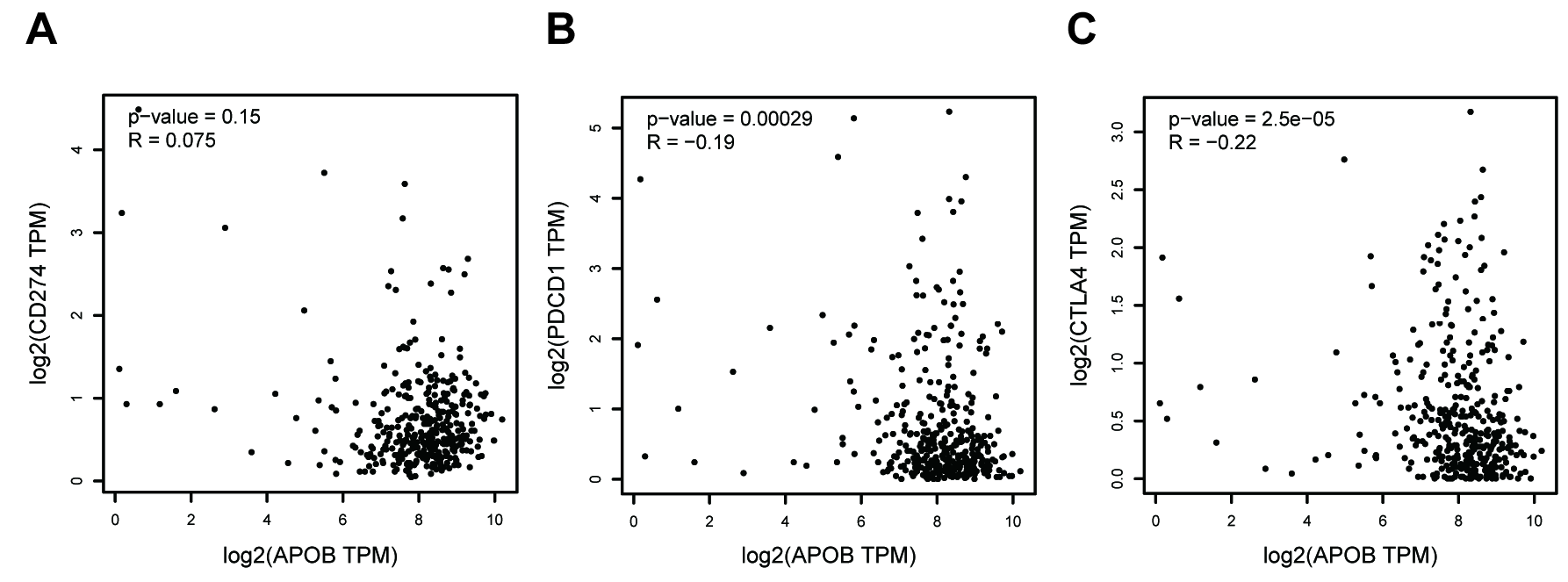


Supplementary Figure 3 Correlation of APOB expression with CD274, PDCD1, and CTLA4 expression in HCC. (A-C) Spearman correlation of APOB with expression of CD274 (A), PDCD1 (B), CTLA-4 (C) in HCC using GEPIA.
