# Supplementary material for: APOB is a potential prognostic biomarker in hepatocellular carcinoma
Source: Discov Oncol. 2024 Feb 3;15:28. doi: 10.1007/s12672-024-00877-6 (PMC10838261; doi:10.1007/s12672-024-00877-6)
Supplement: Supplementary file 2 — Additional file2 (DOCX 20 KB) [file 12672_2024_877_MOESM2_ESM.docx]

Supplementary Table 1 The related lincRNA of hsa-miR-877-5p by starbase database.

| MiRNA ID | MiRNA Name | Gene ID | Gene Name | Gene Type |
| --- | --- | --- | --- | --- |
| MIMAT0000076 | hsa-miR-21-5p | ENSG00000224959 | AC017002.1 | lincRNA |
| MIMAT0000076 | hsa-miR-21-5p | ENSG00000245937 | LINC01184 | lincRNA |
| MIMAT0000076 | hsa-miR-21-5p | ENSG00000273329 | AC078846.1 | lincRNA |
| MIMAT0000076 | hsa-miR-21-5p | ENSG00000271204 | AC016831.5 | lincRNA |
| MIMAT0000076 | hsa-miR-21-5p | ENSG00000225725 | FAM66E | lincRNA |
| MIMAT0000076 | hsa-miR-21-5p | ENSG00000249859 | PVT1 | lincRNA |
| MIMAT0000076 | hsa-miR-21-5p | ENSG00000280798 | LINC00294 | lincRNA |
| MIMAT0000076 | hsa-miR-21-5p | ENSG00000251562 | MALAT1 | lincRNA |
| MIMAT0000076 | hsa-miR-21-5p | ENSG00000250742 | LINC02381 | lincRNA |
| MIMAT0000076 | hsa-miR-21-5p | ENSG00000275409 | AC026367.2 | lincRNA |
| MIMAT0000076 | hsa-miR-21-5p | ENSG00000256204 | AC073862.1 | lincRNA |
| MIMAT0000076 | hsa-miR-21-5p | ENSG00000260073 | AC023824.1 | lincRNA |
| MIMAT0000076 | hsa-miR-21-5p | ENSG00000274220 | AC009163.7 | lincRNA |
| MIMAT0000076 | hsa-miR-21-5p | ENSG00000225975 | LINC01534 | lincRNA |
| MIMAT0000076 | hsa-miR-21-5p | ENSG00000267242 | AC069278.4 | lincRNA |
| MIMAT0000076 | hsa-miR-21-5p | ENSG00000267838 | AC245884.8 | lincRNA |
| MIMAT0000076 | hsa-miR-21-5p | ENSG00000229807 | XIST | lincRNA |
| MIMAT0000076 | hsa-miR-21-5p | ENSG00000230590 | FTX | lincRNA |

Supplementary Table 2 The related lincRNA of hsa-miR-9-5p by starbase database.

| MiRNA ID | MiRNA Name | Gene ID | Gene Name | Gene Type |
| --- | --- | --- | --- | --- |
| MIMAT0000441 | hsa-miR-9-5p | ENSG00000235576 | LINC01871 | lincRNA |
| MIMAT0000441 | hsa-miR-9-5p | ENSG00000163364 | LINC01116 | lincRNA |
| MIMAT0000441 | hsa-miR-9-5p | ENSG00000273301 | AC016717.2 | lincRNA |
| MIMAT0000441 | hsa-miR-9-5p | ENSG00000237940 | LINC01238 | lincRNA |
| MIMAT0000441 | hsa-miR-9-5p | ENSG00000246876 | LINC02466 | lincRNA |
| MIMAT0000441 | hsa-miR-9-5p | ENSG00000272221 | AL645933.2 | lincRNA |
| MIMAT0000441 | hsa-miR-9-5p | ENSG00000242258 | LINC00996 | lincRNA |
| MIMAT0000441 | hsa-miR-9-5p | ENSG00000197308 | GATA3-AS1 | lincRNA |
| MIMAT0000441 | hsa-miR-9-5p | ENSG00000245532 | NEAT1 | lincRNA |
| MIMAT0000441 | hsa-miR-9-5p | ENSG00000246067 | RAB30-AS1 | lincRNA |
| MIMAT0000441 | hsa-miR-9-5p | ENSG00000256155 | AC022075.2 | lincRNA |
| MIMAT0000441 | hsa-miR-9-5p | ENSG00000274292 | AC084018.2 | lincRNA |
| MIMAT0000441 | hsa-miR-9-5p | ENSG00000224511 | LINC00365 | lincRNA |
| MIMAT0000441 | hsa-miR-9-5p | ENSG00000230490 | AL139383.1 | lincRNA |
| MIMAT0000441 | hsa-miR-9-5p | ENSG00000259129 | LINC00648 | lincRNA |
| MIMAT0000441 | hsa-miR-9-5p | ENSG00000214900 | LINC01588 | lincRNA |
| MIMAT0000441 | hsa-miR-9-5p | ENSG00000281005 | LINC00921 | lincRNA |
| MIMAT0000441 | hsa-miR-9-5p | ENSG00000277369 | AC010654.1 | lincRNA |
| MIMAT0000441 | hsa-miR-9-5p | ENSG00000245694 | CRNDE | lincRNA |
| MIMAT0000441 | hsa-miR-9-5p | ENSG00000227036 | LINC00511 | lincRNA |
| MIMAT0000441 | hsa-miR-9-5p | ENSG00000232677 | LINC00665 | lincRNA |
| MIMAT0000441 | hsa-miR-9-5p | ENSG00000279082 | LINC01727 | lincRNA |
| MIMAT0000441 | hsa-miR-9-5p | ENSG00000229807 | XIST | lincRNA |
| MIMAT0000441 | hsa-miR-9-5p | ENSG00000270052 | BX546450.2 | lincRNA |

Supplementary Table 3 The related lincRNA of hsa-miR-877-5p by starbase database.

| MiRNA id | MiRNA name | Gene ID | Gene Name | Gene Type |
| --- | --- | --- | --- | --- |
| MIMAT0004949 | hsa-miR-877-5p | ENSG00000198468 | FLVCR1-AS1 | lincRNA |
| MIMAT0004949 | hsa-miR-877-5p | ENSG00000204792 | LINC01291 | lincRNA |
| MIMAT0004949 | hsa-miR-877-5p | ENSG00000222041 | CYTOR | lincRNA |
| MIMAT0004949 | hsa-miR-877-5p | ENSG00000227403 | LINC01806 | lincRNA |
| MIMAT0004949 | hsa-miR-877-5p | ENSG00000242086 | MUC20-OT1 | lincRNA |
| MIMAT0004949 | hsa-miR-877-5p | ENSG00000224652 | LINC00885 | lincRNA |
| MIMAT0004949 | hsa-miR-877-5p | ENSG00000259959 | AC107068.1 | lincRNA |
| MIMAT0004949 | hsa-miR-877-5p | ENSG00000246876 | LINC02466 | lincRNA |
| MIMAT0004949 | hsa-miR-877-5p | ENSG00000272335 | AC093297.2 | lincRNA |
| MIMAT0004949 | hsa-miR-877-5p | ENSG00000152931 | PART1 | lincRNA |
| MIMAT0004949 | hsa-miR-877-5p | ENSG00000245556 | SCAMP1-AS1 | lincRNA |
| MIMAT0004949 | hsa-miR-877-5p | ENSG00000273142 | AC073335.2 | lincRNA |
| MIMAT0004949 | hsa-miR-877-5p | ENSG00000255366 | AC120036.4 | lincRNA |
| MIMAT0004949 | hsa-miR-877-5p | ENSG00000259820 | AC083843.3 | lincRNA |
| MIMAT0004949 | hsa-miR-877-5p | ENSG00000281649 | EBLN3P | lincRNA |
| MIMAT0004949 | hsa-miR-877-5p | ENSG00000197308 | GATA3-AS1 | lincRNA |
| MIMAT0004949 | hsa-miR-877-5p | ENSG00000273038 | AL365203.2 | lincRNA |
| MIMAT0004949 | hsa-miR-877-5p | ENSG00000245532 | NEAT1 | lincRNA |
| MIMAT0004949 | hsa-miR-877-5p | ENSG00000212694 | LINC01089 | lincRNA |
| MIMAT0004949 | hsa-miR-877-5p | ENSG00000247373 | AC055713.1 | lincRNA |
| MIMAT0004949 | hsa-miR-877-5p | ENSG00000258630 | LINC02292 | lincRNA |
| MIMAT0004949 | hsa-miR-877-5p | ENSG00000277782 | AC068870.2 | lincRNA |
| MIMAT0004949 | hsa-miR-877-5p | ENSG00000261241 | LINC02128 | lincRNA |
| MIMAT0004949 | hsa-miR-877-5p | ENSG00000269947 | AC135178.5 | lincRNA |
| MIMAT0004949 | hsa-miR-877-5p | ENSG00000188825 | LINC00910 | lincRNA |
| MIMAT0004949 | hsa-miR-877-5p | ENSG00000265282 | AC005828.4 | lincRNA |
| MIMAT0004949 | hsa-miR-877-5p | ENSG00000272688 | AP005329.3 | lincRNA |
| MIMAT0004949 | hsa-miR-877-5p | ENSG00000263753 | LINC00667 | lincRNA |
| MIMAT0004949 | hsa-miR-877-5p | ENSG00000267100 | ILF3-AS1 | lincRNA |
| MIMAT0004949 | hsa-miR-877-5p | ENSG00000267886 | AC074135.1 | lincRNA |
| MIMAT0004949 | hsa-miR-877-5p | ENSG00000268568 | AC007228.2 | lincRNA |
| MIMAT0004949 | hsa-miR-877-5p | ENSG00000268333 | AL132655.1 | lincRNA |
| MIMAT0004949 | hsa-miR-877-5p | ENSG00000276786 | BX640515.1 | lincRNA |
| MIMAT0004949 | hsa-miR-877-5p | ENSG00000270012 | AC232271.1 | lincRNA |
| MIMAT0004949 | hsa-miR-877-5p | ENSG00000229807 | XIST | lincRNA |
